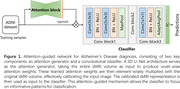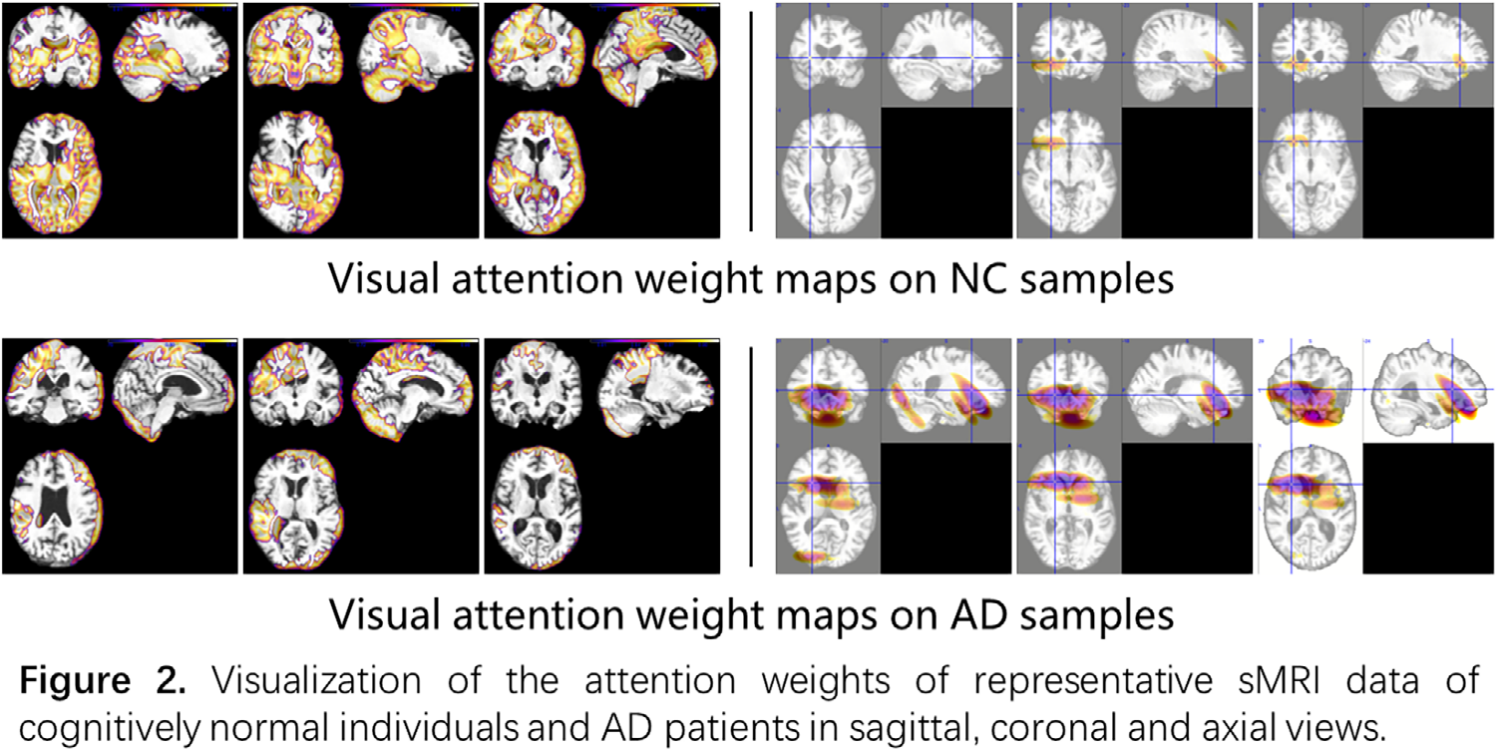# Attention‐Guided Deep Learning Discovers Brain Morphological Patterns of Alzheimer’s Disease from Structural MRI

**DOI:** 10.1002/alz.095663

**Published:** 2025-01-09

**Authors:** Ran Gu, Hongming Li, Yong Fan

**Affiliations:** ^1^ University of Pennsylvania, Philadelphia, PA USA; ^2^ Perelman School of Medicine, University of Pennsylvania, Philadelphia, PA USA

## Abstract

**Background:**

Structural Magnetic Resonance Imaging (sMRI) is an invaluable tool for detecting brain morphological changes to aid in the diagnosis of Alzheimer’s Disease (AD). However, the heterogeneity of AD‐related neurodegeneration poses significant challenges for conventional image analysis techniques to accurately capture discriminative brain changes from whole‐brain sMRI data.

**Method:**

We present a novel attention‐guided deep learning framework to automatically identify and focus on the most discriminative brain regions for robust AD diagnosis from sMRI scans (Figure 1). Our model leverages a 3D U‐Net as an attention generator to learn voxel‐wise attention weights to calibrate the original sMRI data via an attention mechanism, and the calibrated representation is subsequently passed to a classifier for AD diagnosis. This attention‐guided process enables the model to selectively focus on the most informative brain regions for feature learning and decision‐making. Our model has been evaluated on the ADNI dataset.

**Result:**

A deep learning model was trained on the ADNI‐1 data and evaluated on the ADNI Go & 2 data. The model achieved an accuracy of 90.0%, precision of 82.3%, recall of 87.3%, and F1‐score of 84.7% in discriminating AD patients from cognitively normal individuals using sMRI data. The accuracy was better than an alternative model without the non‐attention‐guided model (with an accuracy of 87.6%), demonstrating the effectiveness of the attention‐guided mechanism. Visualization of the learned attention maps revealed salient regions localized in the hippocampus, ventricles, temporal lobes, and other areas known to be affected by AD (Figure 2).

**Conclusion:**

The proposed attention‐guided deep learning framework demonstrated superior performance compared to traditional methods for AD diagnosis from sMRI scans. By explicitly learning to focus on discriminative brain regions, our model effectively captures AD related brain morphological patterns.